# Dietary ferulic acid supplementation improved cottonseed meal-based diet utilization by enhancing intestinal physical barrier function and liver antioxidant capacity in grass carp (*Ctenopharyngodon Idellus*)

**DOI:** 10.3389/fphys.2022.922037

**Published:** 2022-08-22

**Authors:** Shiyou Chen, Yan Lin, Hequn Shi, Linghong Miao, Bo Liu, Xianping Ge

**Affiliations:** ^1^ Wuxi Fisheries College, Nanjing Agricultural University, Wuxi, China; ^2^ Key Laboratory of Freshwater Fisheries and Germplasm Resources Utilization, Ministry of Agriculture, Freshwater Fisheries Research Center, Chinese Academy of Fishery Sciences, Wuxi, China; ^3^ National Demonstration Center for Experimental Fisheries Science Education, Shanghai Ocean University, Shanghai, China; ^4^ Guangzhou Cohoo Bio-tech Research & Development Centre, Guangzhou, China

**Keywords:** cottonseed meal, ferulic acid, grass carp, antioxidation, growth performance, tight junction

## Abstract

The present study explored the effects of ferulic acid (FA) supplementation in cottonseed meal (CSM)-based diets on grass carp growth performance, feed utilization, liver antioxidation status, and intestinal physical barrier function. Here, four experimental diets supplemented with FA at graded levels (0, 50, 100 and 200 mg/kg) and CSM as the main protein source (384.6 g/kg feed) for an 8-week feeding trial. Our results indicated that 200 mg/kg FA supplementation in a CSM-based diet significantly improved growth performance [including final body weight (FBW), weight gain rate, and specific growth rate] and feed utilization [including feed conversion ratio and protein efficiency ratio] in grass carp (*p* < 0.05). The results of polynomial regression analysis based on FBW recommended that the optimal dose for FA supplementation was 204 mg/kg. Compared with that no FA supplementation, 200 mg/kg FA supplementation significantly reduced liver malondialdehyde levels and increased glutathione reductase activities (*p* < 0.05) and 100 mg/kg FA supplementation significantly increased liver total superoxide dismutase activities and reduced blood alanine transaminase levels (*p* < 0.05). Compared with the control group, 100 mg/kg FA supplementation also led to significantly increased mRNA expression of *zo-1*, *zo-2*, *occludin*, *claudin-b*, *claudin-3*, *claudin-7a*, and *claudin-12*, encoding intestinal tight junction proteins (*p* < 0.05). Notably, FA supplementation could reduce lipid deposition by regulating bile acid (BA) secretion. In this study, 100 and 200 mg/kg FA supplementation significantly increased blood and liver total BA levels, respectively (*p* < 0.05); 100 mg/kg FA also significantly activated mRNA expressions of *fxr* and *cyp7a1* (*p* < 0.05). Furthermore, the whole-body composition results presented that FA treatment relieved lipid deposition, particularly 50 and 200 mg/kg FA supplementation (*p* < 0.05). Moreover, triglyceride and total cholesterol levels were significantly lower and high-density lipoprotein levels were significantly higher with 200 mg/kg FA supplementation than with no FA supplementation (*p* < 0.05). Taken together, the results indicated that FA may be a beneficial feed additive to boost fish growth performance and increase CSM utilization.

## Highlights

1) Ferulic acid (FA) supplementation improved cottonseed meal (CSM)-based diet utilization.
2) FA supplementation improved growth performance.
3) FA supplementation in CSM-based diets improved intestine physical barrier function.
4) FA supplementation in CSM-based diets improved liver antioxidant capacity.
5) FA improved lipid metabolism by regulating bile acid secretion.

## Introduction

Over decades, the application of plant proteins in aquatic feed has attracted considerable research attention, partly due to the limited resources and high costs of fishmeal (FM) (Tacon and Metian, 2008; Mokrani et al., 2019; Liu X. et al., 2020; Henry et al., 2020; Menegasso et al., 2020; Li et al., 2021). Of the plant proteins, cottonseed meal (CSM) is considered to have great potential as an FM substitute in aquatic feed (Bian et al., 2017; Huang et al., 2017). This is because CSM is resource-abundant and more cost-effective than FM and soybean meal (SBM) (Li and Robinson, 2006; Bu et al., 2017; Delgado et al., 2021). A considerably high amount of CSM is produced in China—second only to that in India globally (Index Mundi, 2021). Increasing CSM proportion in aquatic formulation feed may facilitate the sustainable development of aquaculture. However, the presence of antinutritional factors (ANFs), such as free gossypol (FG), in CSM has greatly limited CSM’s use in aquatic feed (Wang et al., 2014; Wang T. et al., 2020). Grass carp (*Ctenopharyngodon idellus*) (Zheng et al., 2012), African catfish (*Clarias gariepinus*) (Imorou Toko et al., 2008), tilapia (*Oreochroms mossambcus*) (Mbahinzireki et al., 2001; Garcia-Abiado et al., 2004), and common carp (*Cyprinus carpio*) (Wang et al., 2014) fed with CSM-based diets have been found to have negative effects on their growth and health. Moreover, FG that exists in the feed may trigger oxidative damage and impair the intestinal physical barrier, affecting absorption capacity (Wang et al., 2018; Wang K. Z. et al., 2019). In practice, antibiotic growth promoters are often used for resolving the above-mentioned problems induced by ANFs (Liu Y. L. et al., 2020). However, this increases the risks of antibiotic residue, food safety, and antimicrobial resistance and thus violates the “green aquaculture” concept (Ringø et al., 2015). Consequently, the use of herbal medicines attracts a considerable amount of attention for sustainable aquaculture development because its advantages include wide distribution, low toxicity, limited adverse effects, and lack of drug resistance and residue (Pu et al., 2017; Hoseinifar et al., 2020; Zhu, 2020).

**FIGURE 1:**
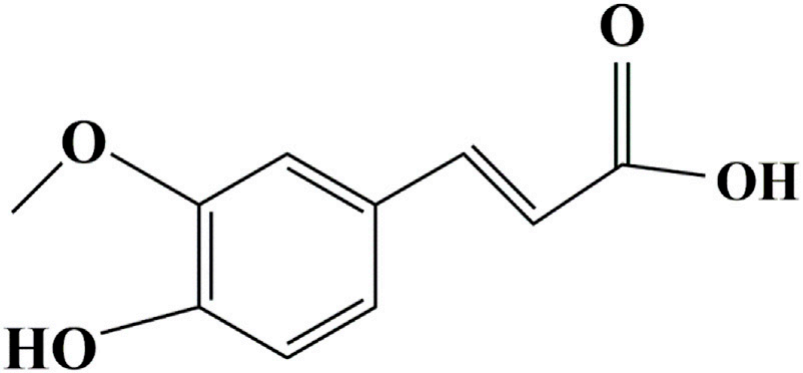
Molecular structure of ferulic acid.

Ferulic acid (FA), the extract of *Angelica sinensis*, *Ligusticum chuanxiong* Hort, and *Cimicifuga heracleifolia*, has been widely studied in recent decades in the pharmaceutical, food, and cosmetics industries due to the characteristics of antioxidant, antibacterial, anti-inflammatory, antihyperlipidemic, growth-promoting, and immunostimulant properties (Mathew and Abraham, 2004; Kumar and Pruthi, 2014; Zdunska et al., 2018). FA exerts resistance tolerance to oxidative stress or inflammation caused by cadmium chloride (CdCl_2_) (Kassab et al., 2020), ultraviolet B radiation (Ambothi et al., 2015), and diabetes (Ghosh et al., 2018) or hyperglycemia (Chowdhury et al., 2019). These properties are partly attributable to FA’s particular structural motifs, the 3-methoxy and 4-hydroxyl, and the carboxylic acid groups (Graf, 1992; Kanski et al., 2002) (the structure presented in Figure 1). Gorewit (1983) attributed FA’s growth-enhancing properties to the structure resembling normetanephrine, which stimulated somatotropin secretion in the pituitary gland. FA is considered a functional feed additive with antistress or anti-inflammatory properties in aquatic feed as well. Our previous research (Chen et al., 2021) confirmed that FA could alleviate inflammation and oxidative damage from a lipopolysaccharide (LPS) challenge in blunt snout bream (*Megalobrama amblycephala*). Yu et al. (2020) also elucidated that dietary FA supplementation can counteract oxidative damage induced by oxidized fish oil in tilapia. FA can also be a growth stimulant in aquaculture (Yu et al., 2017; Ahmadifar et al., 2019; Dawood et al., 2020). In a *Nannochloropsis oculata* culture, FA addition (100 mg/L) has been shown to contribute to 2.52- and 2.02-fold increases in cell density and specific growth rate (SGR), respectively (Tam et al., 2020). Dietary supplementation of 100 mg/kg FA was reported to significantly decrease feed conversion ratio (FCR) and increase the weight gain rate (WGR) of tilapia (Yu et al., 2018). Fu et al. (2022) exhibited that juvenile hybrid grouper (*Epinephelus fuscoguttatus*♀ × *Epinephelus polyphekadion*♂) fed the diets with FA supplementation of 80 mg/kg presented significantly improved growth performance and feed utilization.

Grass carp, the most popularly farmed freshwater fish worldwide, has a great commercial value (Wang et al., 2015; Qin et al., 2020). In 2018, farmed grass carp production was more than 5.7 million tons, accounting for 10.5% of the total finfish production globally (FAO, 2020). Studies (Wang et al., 2018; Wang K. Z. et al., 2019) have reported that ANFs (especially FG) in CSM impaired growth performance, damaged intestinal structure, aggravated inflammation, and reduced intestinal immunity and absorption capacity in grass carp. However, the addition of herbal products has been found to improve the growth performance and immune responses of grass carp fed CSM-based and rapeseed meal-based diets (Liu Y. L. et al., 2020). Therefore, in the present study, we evaluated the effects of FA supplementation in CSM-based diets on the growth performance, the liver antioxidant status, and the intestinal physical barrier function of grass carp.

## Materials and methods

### Diets’ preparation

Based on previous studies (Zheng et al., 2012; Wang et al., 2018; Wang K. Z. et al., 2019; Chen et al., 2021), the experimental diets, CSM (containing 1,275 mg/kg FG) as the main protein source, supplemented with graded levels of FA (0, 50, 100 and 200 mg/kg, Dalian Meilun Biological Technology, Dalian, China) were formulated; the CSM–based diets used here contained nearly 400 mg/kg FG. Detailed formula and measured nutrients were provided in Table 1. The feed-making process was based on the protocol reported in our previous publication (Jingyuan et al., 2020). After drying it naturally, we packed the experimental diets in labelled sealed bags and stored them at −15°C for further use.

**TABLE 1:** Ingredient and nutrient composition of experimental diets.

Ingredient	Diets (g dry matter)			
	Control	FA (50)	FA (100)	FA (200)
Fish meal	53.3	53.3	53.3	53.3
Soybean meal	64.8	64.8	64.8	64.8
Cottonseed meal	384.6	384.6	384.6	384.6
Cottonseed protein concentrate	84.5	84.5	84.5	84.5
Wheat flour	177.2	177.2	177.2	177.2
Rice bran	120.1	120.1	120.1	120.1
Wheat bran	49.5	49.5	49.5	49.5
Soybean oil	20	20	20	20
Calcium dihydrogen phosphate	10	10	10	10
Mineral premix^a^	5	5	5	5
Vitamins premix^b^	5	5	5	5
Vitamin C (95%)	5	5	5	5
Choline chloride	5	5	5	5
Microcrystalline cellulose	10	9.95	9.9	9.8
Bentonite	6	6	6	6
Ferulic acid	0	0.05	0.1	0.2
Total	1,000	1,000	1,000	1,000
Nutrient contents (dry matter)^c^				
Crude protein (%)	39.67	39.52	39.44	39.65
Crude lipid (%)	7.24	6.97	6.65	6.83
Ash (%)	8.66	8.70	9.01	8.75
Gross energy (MJ/kg)	19.60	19.80	19.70	19.71
Free gossypol (mg/kg)	406.20	398.16	417.94	406.81

Note: The crude protein of fish meal, soybean meal, cottonseed meal, cottonseed protein concentrate used in the study was 69.5%, 46.37%, 55.6%, and 61.5%, respectively. Meanwhile, the crude lipid of these protein source was 9.46%, 4.25%, 4.49%, and 2.36%, respectively. Nearly 1,275 mg/kg free gossypol was detected in the cottonseed meal.

a Mineral premix (IU, g or mg/kg of diet): calcium biphosphate, 20 g; sodium chloride, 2.6 g; potassium chloride, 5 g; magnesium sulfate, 2 g; ferrous sulfate, 0.9 g; zinc sulfate, 0.06 g; cupric sulfate, 0.02 g; manganese sulfate, 0.03 g; sodium selenate, 0.02 g; cobalt chloride, 0.05 g; potassium iodide, 0.004 g

b Vitamins premix (IU, g or mg/kg of diet): vitamin A, 25,000 IU; vitamin D3, 20,000 IU; vitamin E, 200 mg; vitamin K3, 20 mg; thiamin, 40 mg; riboflavin, 50 mg; calcium pantothenate, 100 mg; pyridoxine HCl, 40 mg; cyanocobalamin, 0.2 mg; biotin, 6 mg; folic acid, 20 mg; niacin, 200 mg; inositol, 1,000 mg; vitamin C, 2000 mg; choline, 2000 mg.

c All are measured values.

### Fish and feeding trial

Juvenile grass carp (*Ctenopharyngodon idellus*) were obtained from the Nanquan fish hatcheries affiliated to the Freshwater Fisheries Research Center (Wuxi, China) and acclimated for 2 weeks in an outdoor cage (4 m × 4 m × 1 m). During this time, the fish were fed three times daily with commercial diets (38% crude protein and 7% crude fat). After acclimation, 360 healthy juvenile grass carp with similar weights (initial average body weight = 5.0 ± 0.5 g per juvenile) were randomly distributed into 12 floating net cages (1 m × 1 m × 1 m) with a stocking capacity of 30 fish per cage. Moreover, we ensured that the total body weight of 30 fish per cage was 150 g, avoiding differences in initial fish weight between groups.

The four experimental diets were randomly assigned to these net cages in triplicate. Fish were hand-fed to apparent satiation three times daily (at 7.30 a.m., 11.30 a.m., and 5.00 p.m.) for 8 weeks. The whole feeding trial was performed in an outdoor pond under natural light conditions, and water temperature and pH were 26.5–32.4°C and 6.8–7.4, respectively. In addition, the rotary impeller aerator was run for 6 h per day to ensure enough dissolved oxygen during the feeding trial.

### Sampling and chemical analyses

At the end of the 8-week feeding trial, the fish were starved for 36 h before being sampled. The weight and number of fish per cage were measured for growth performance and survival rate indices analysis. Thereafter, four fish were randomly collected from each cage and then stored at −20°C for the analysis of whole-body nutrient chemical composition. Another three fish were randomly collected from each cage for blood, liver, and intestine sampling. Blood samples obtained from the caudal vein were collected in anticoagulant tubes first and then centrifuged at 4,000 × *g* for 10 min at 4°C. The supernatant plasma was then collected and stored at −20°C for blood parameters determination. After blood collection, the abdominal cavity of the fish was cut and opened immediately. The liver and intestine were separated, sampled, and stored at −80°C for enzyme activity, total bile acid (TBA) level, and gene expression measurements.

The chemical compositions of feed and fish were determined using the AOAC (2003) methods. In brief, samples were dried at 105°C to constant weight for moisture measurement and then combusted in a muffle furnace at 550°C lasting 5 h for crude ash analysis. Crude protein was obtained using the Kjeldahl method (N × 6.25), lipid using a Soxhlet extractor (petroleum ether extraction), and gross energy using an oxygen bomb calorimeter (combustion method). The FG contents in diets were measured according to the Chinese national standard GB/T 13086-2020.

Liver enzyme activities, such as total antioxidant capacity (T-AOC) and total superoxide dismutase (T-SOD), catalase (CAT), glutathione s-transferase (GST), glutathione reductase (GR), glutathione (GSH), and malondialdehyde (MDA) levels, were tested by kits purchased from Nanjing Jiancheng Bioengineering Institute (Nanjing, China). Plasma aspartate transaminase (AST), alanine transaminase (ALT), triglyceride (TG), low-density lipoprotein (LDL), total cholesterol (TC), and high-density lipoprotein (HDL) were determined on Mindray BS-400 (Mindray Medical International, Shenzhen, China). The kits used here were obtained from Shanghai Zhicheng Biological Technology. Blood and liver TBA levels were measured using kits from Jiancheng Bioengineering Institute (Nanjing, China) according to the manufacturer’s instructions.

### RNA extraction and real-time reverse transcription quantitative polymerase chain reaction

Total mRNAs in the intestine and liver tissues were extracted using RNAiso plus kit (TaKaRa, China), and then, the mRNA concentration was measured using a Nanodrop system (DN-2000; Thermo Scientific). cDNA was synthesized using HiScript III RT SuperMix cDNA synthesis kits (Vazyme Biotech, Nanjing, China). Real-time reverse transcription quantitative polymerase chain reaction (RT-qPCR; total volume = 20 µl) was performed on a Bio-Rad CFX96 Real-Time PCR System using ChamQ Universal SYBR qPCR Master Mix kit (Vazyme Biotech, Nanjing, China). The program for qPCR herein was as follows: 95°C for 30 s (pre-denaturation); 95°C for 5 s (denaturation), followed by 60°C for 30 s (40 cycles); finally, 95°C for 10 s. RT-qPCR–specific primers used here have been designed in previous studies (Xu et al., 2016; Wei et al., 2020) and synthesized by Shanghai Biological Engineering (Table 2). The relative mRNA expression was determined using the 2^−ΔΔCt^ method.

**TABLE 2:** RT-qPCR primers used for relative mRNA expression analyses.

Gene name	Sequences (5′→3′)	GenBank no.	References
*fxr*	F: GCA​GAG​TGC​CTG​CTT​ACA​GA	KT861862	
	R: GAG​CAG​TGC​GTG​TTG​TCT​TG		
*cyp7a1*	F: TCT​ATG​ACA​ATC​CTC​TGG​CAT​ACA​A	KT831860	
	R: CAA​AGA​AAC​GGC​CCG​GAC​A		
*zo-1*	F: CGGTGTCTTCGTAGTCGG	KJ000055	Xu et al. (2016)
	R: CAG​TTG​GTT​TGG​GTT​TCA​G		
*zo-2*	F: TAC​AGC​GGG​ACT​CTA​AAA​TGG	KM112095	Xu et al. (2016)
	R: TCA​CAC​GGT​CGT​TCT​CAA​AG		
*jam-a*	F: ACTGTGAGGTGCTTGGAA	KY780630	Wei et al. (2020)
	R: CTG​TTG​TGA​CTG​AAG​AAG​GA		
*occludin*	F: TAT​CTG​TAT​CAC​TAC​TGC​GTC​G	KF193855	Xu et al. (2016)
	R: CATTCACCCAATCCTCCA		
*claudin-b*	F: GAGGGAATCTGGATGAGC	KF193860	Xu et al. (2016)
	R: ATGGCAATGATGGTGAGA		
*claudin-c*	F: GAGGGAATCTGGATGAGC	KF193859	Xu et al. (2016)
	R: CTGTTATGAAAGCGGCAC		
*claudin-3*	F: ATCACTCGGGACTTCTA	KF193858	Xu et al. (2016)
	R: CAGCAAACCCAATGTAG		
*claudin-7a*	F: ACT​TAC​CAG​GGA​CTG​TGG​ATG​T	KT625604	Xu et al. (2016)
	R: CAC​TAT​CAT​CAA​AGC​ACG​GGT		
*claudin-12*	F: CCCTGAAGTGCCCACAA	KF998571	Xu et al. (2016)
	R: GCGTATGTCACGGGAGAA		
*claudin-15a*	F: TGC​TTT​ATT​TCT​TGG​CTT​TC	KF193857	Xu et al. (2016)
	R: CTC​GTA​CAG​GGT​TGA​GGT​G		
*β-actin*	F: GGCTGTGCTGTCCCTGTA	M25013	Xu et al. (2016)
	R: GGG​CAT​AAC​CCT​CGT​AGA​T		

Note: Reverse transcription quantitative PCR (RT-qPCR), forward primer (F), reverse primer (R), farnesoid X receptor (*fxr*), cholesterol 7α-hydroxylase (*cyp7a1*), zonula occludens-1/2 (*zo-1*/*2*), junctional adhesion molecule-A (*jam-a*).

### Statistical analyses

Statistical analyses were performed on SPSS (version 20.0; SPSS Inc., Chicago, IL, United States) and GraphPad Prism (version 8.0; GraphPad Software, San Diego, CA, United States). All data were reviewed for normality and homogeneity of variance using the Shapiro–Wilk test and Levene’s test, respectively, followed by one-way analysis of variance and Duncan’s multiple comparisons. All data are reported as means ± standard errors of the means. A *p* value of < 0.05 was considered to represent significant differences.

## Results

### Growth performance parameters

**FIGURE 2:**
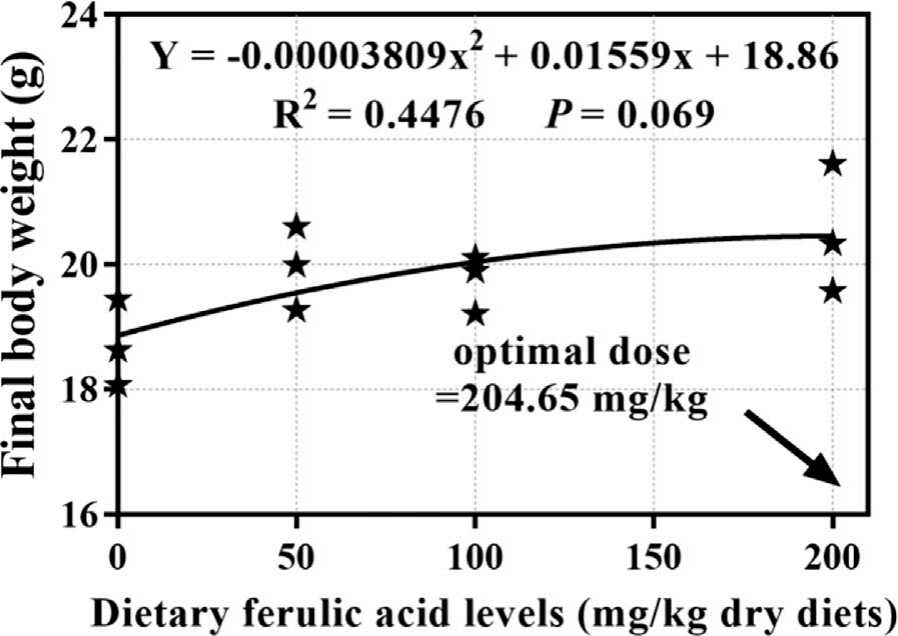
Quadratic regression analysis of dietary ferulic acid levels versus final body weight in juvenile grass carp.

During the 8-week feeding trial, there was only one dead fish found in the group fed 50 mg/kg FA-supplemented diet, and the indicator of survival rate (SR; *p* > 0.05) showed no significant difference between the groups. As shown in Table 3, the differences between the four treatment groups in final body weight (FBW), weight gain rate (WGR), SGR, protein efficiency ratio (PER), and feed conversion ratio (FCR) were significant *p* < 0.05. In detail, after 8 weeks of feeding, the FBW, WGR, and SGR of grass carp were highest in the group fed a 200 mg/kg FA-supplemented diet, and they significantly differed from those in the control group (*p* < 0.05). Moreover, the 200 mg/kg FA-supplemented diet improved feed efficiency: The FCR decreased and the PER increased significantly compared with the control group (*p* < 0.05). Furthermore, 50 and 100 mg/kg FA-supplemented diets had a positive effect on the FBW, WGR, SGR, and PER increase and FCR decrease, but there was no significant difference compared with the control group (*p* > 0.05). The polynomial regression analysis based on FBW (Figure 2) indicated that the optimal FA supplementation doses in CSM-based diets were 204 mg/kg.

**TABLE 3:** Growth performance of juvenile grass carp fed diets containing graded levels of FA.

Growth performance	Groups			
	Control	FA (50)	FA (100)	FA (200)
FBW (g/fish)^a^	18.71 ± 0.40^a^	19.95 ± 0.38^ab^	19.74 ± 0.27^ab^	20.51 ± 0.59^b^
WGR (%)^b^	274.27 ± 7.97^a^	299.07 ± 7.67^ab^	294.8 ± 5.47^ab^	310.18 ± 11.82^b^
SGR (% day ^−1^)^c^	2.27 ± 0.04^a^	2.39 ± 0.03^ab^	2.37 ± 0.02^ab^	2.43 ± 0.05^b^
FCR^d^	1.66 ± 0.05^b^	1.55 ± 0.03^ab^	1.55 ± 0.05^ab^	1.46 ± 0.05^a^
PER^e^	1.52 ± 0.04^a^	1.63 ± 0.03^ab^	1.64 ± 0.05^ab^	1.73 ± 0.07^b^
SR (%)^f^	100.00 ± 0.00	98.89 ± 1.11	100.00 ± 0.00	100.00 ± 0.00

Note: All data presented as means of three replicates ±standard error of the means. Means in the same row with different superscripts are significantly different (*p* < 0.05).

a FBW: final body weight.

b Weight gain rate (WGR): = 100 × [(FBW (g) − initial body weight (g))/initial body weight (g)].

c Specific growth rate (SGR): = 100 × [(In (FBW (g)) − In (initial body weight (g)))/days].

d Feed conversion ratio (FCR): = dry feed fed (g)/(FBW (g) − initial body weight (g)).

e Protein efficiency ratio (PER) = (FBW (g) − initial body weight (g))/protein consumption (g).

f Survival rate (SR): = 100 × (final fish number/initial fish number).

### Whole-body composition

The differences in the contents of moisture, crude protein, and ash, but not that of lipid, in the juvenile grass carp fed with CSM-based diets containing graded levels of FA were nonsignificant (*p* > 0.05). The FA-supplemented diets reduced the whole-body lipid contents in the fish (*p* < 0.05). Fish fed 50 and 200 mg/kg FA-supplemented diets showed significantly lower lipid levels than the control group (*p* < 0.05; Table 4).

**TABLE 4:** Whole-body composition of juvenile grass carp fed diets containing graded levels of FA.

Whole-body composition	Groups			
	Control	FA (50)	FA (100)	FA (200)
Moisture (%)	73.62 ± 0.27	73.86 ± 0.87	73.65 ± 0.46	73.69 ± 0.15
Crude protein (%)	14.92 ± 0.31	15.37 ± 0.57	14.66 ± 0.33	15.22 ± 0.16
Lipid (%)	9.14 ± 0.21^b^	8.22 ± 0.40^a^	8.83 ± 0.02^ab^	8.26 ± 0.10^a^
Ash (%)	2.69 ± 0.09	2.71 ± 0.05	2.76 ± 0.19	2.68 ± 0.12

Note: All data presented as means of three replicates ±standard error of the means. Means in the same row with different superscripts are significantly different (*p* < 0.05).

### Plasma lipids parameters

**FIGURE 3:**
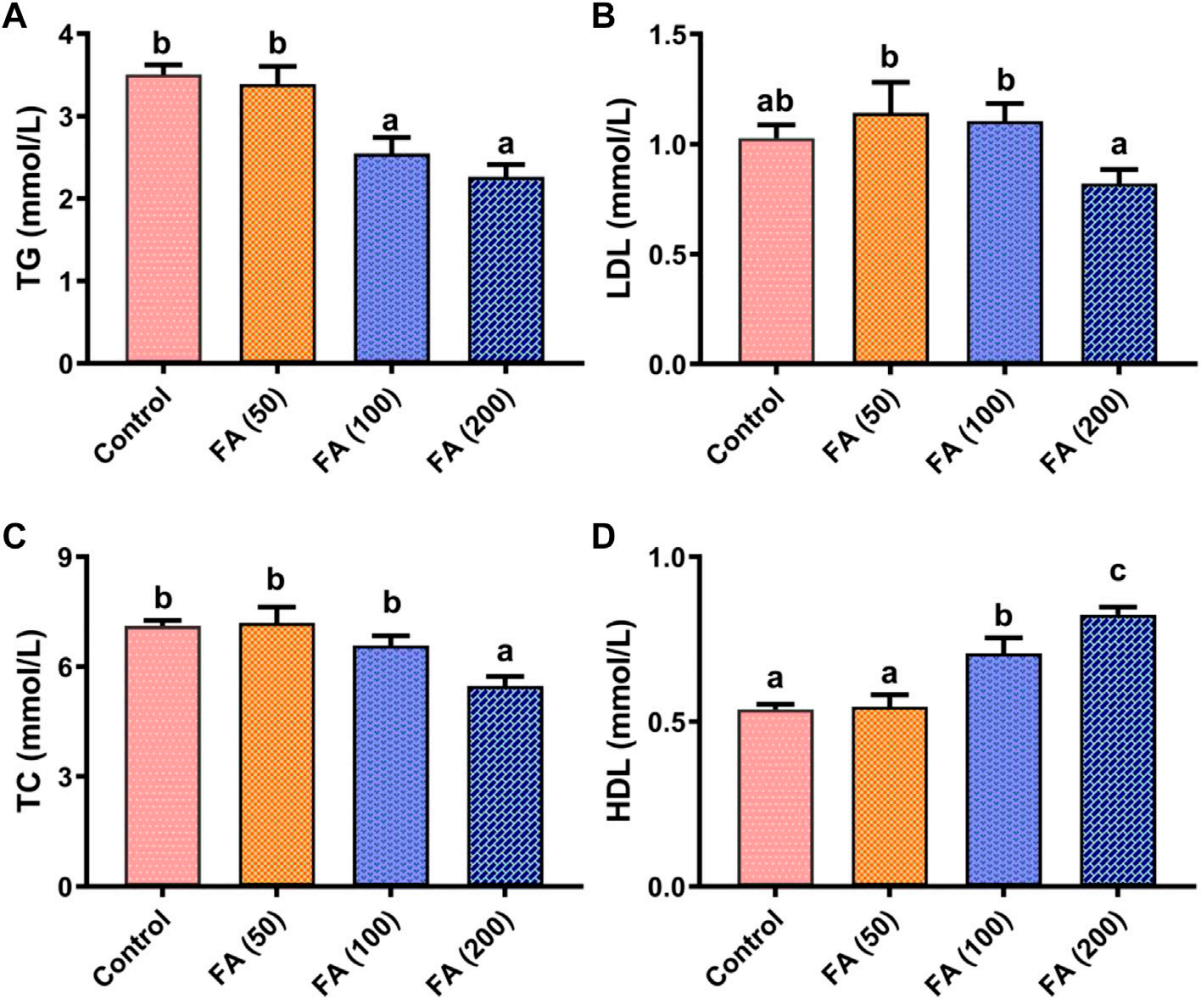
Blood biochemical parameters including triglyceride (TG; **(A)**, low-density lipoprotein (LDL; **(B)**, total cholesterol (TC; **(C)**, and high-density lipoprotein (HDL; **(D)** in grass carp fed CSM-based diets containing graded levels of ferulic acid. Control: high CSM diet; FA (50): high CSM diet +50 mg/kg ferulic acid; FA (100): high CSM diet +100 mg/kg ferulic acid; FA (200): high CSM diet +200 mg/kg ferulic acid. The results are expressed as means ± standard error of the means (*n* = 9). Means of the same parameter with different superscripts are significantly different (*p* < 0.05).

As shown in Figure 3, blood TG (Figure 3A), LDL (Figure 3B), and TC (Figure 3C) levels in grass carp presented a decreasing trend as the levels of FA supplementation increased, whereas HDL levels (Figure 3D) showed an opposite trend. Compared with the control group, 200 mg/kg FA demonstrated lower levels of TG (*p* < 0.05), TC (*p* < 0.05), and LDL (*p* > 0.05). Blood HDL levels in fish fed with 100 and 200 mg/kg FA-supplemented diets were found to be significantly higher than that in fish in the control group and those fed with 50 mg/kg FA (*p* < 0.05). Notably, the highest HDL levels were found in fish fed with 200 mg/kg FA-supplemented diets (*p* < 0.05). However, no remarkable difference was observed in TG, LDH, TC, and HDL between the fish fed with 50 mg/kg FA-supplemented diets and the control group.

### Liver antioxidant abilities and blood indicators reflecting liver function

The results of indicators reflecting the liver antioxidant abilities and liver health status of juvenile grass carp fed CSM-based diets containing graded levels of FA are listed in Table 5. Dietary FA supplementation reduced liver MDA and blood ALT levels. The fish fed with 50 and 100 mg/kg FA-supplemented diets demonstrated lower ALT levels than the control group (*p* < 0.05). In addition, liver MDA levels in fish fed with 50 and 200 mg/kg FA-supplemented diets were significantly lower than that in the control group (*p* < 0.05). 100 and 200 mg/kg FA supplementation [FA (100) and FA (200) groups] significantly enhanced the hepatic enzyme activities of GR and T-SOD than the control group (*p* < 0.05). No significant differences were observed in liver T-AOC, GSH, CAT, GST levels and blood AST levels among the treatment groups (*p* > 0.05).

**TABLE 5:** Indicators reflecting liver antioxidant capacities and liver health in juvenile grass carp fed diets containing graded levels of FA.

Items	Groups			
	Control	FA (50)	FA (100)	FA (200)
In liver				
MDA (nmol/mgprot)	1.15 ± 0.15^b^	0.62 ± 0.06^a^	1.14 ± 0.29^b^	0.58 ± 0.11^a^
T-AOC (mmol/gprot)	0.13 ± 0.02	0.10 ± 0.01	0.12 ± 0.02	0.12 ± 0.02
GR (U/gprot)	3.06 ± 0.84^a^	3.91 ± 1.05^ab^	5.80 ± 1.42^ab^	6.86 ± 1.36^b^
GSH (mg/mgprot)	8.60 ± 0.82	8.47 ± 0.90	8.95 ± 1.37	9.52 ± 2.68
CAT (U/mgprot)	43.54 ± 4.38	42.8 ± 4.56	55.09 ± 5.75	42.84 ± 3.10
GST (U/mgprot)	15.74 ± 3.89	17.83 ± 4.55	9.62 ± 1.48	14.34 ± 3.37
T-SOD (U/mgprot)	121.18 ± 7.27^a^	141.32 ± 14.3^ab^	181.67 ± 17.79^b^	146.32 ± 10.47^ab^
In blood				
AST (U/L)	5.15 ± 0.38	4.66 ± 0.16	5.37 ± 0.54	4.67 ± 0.34
ALT (U/L)	154.50 ± 11.22^b^	116.53 ± 1.74^a^	124.88 ± 2.57^a^	137.66 ± 5.54^ab^

Note: All data presented as means ± standard error of the means (*n* = 9). Means in the same row with different superscripts are significantly different (*p* < 0.05). Malondialdehyde (MDA), total antioxidant capacity (T-AOC), glutathione reductase (GR), glutathione (GSH), catalase (CAT), glutathione S-transferase (GST), total superoxide dismutase (T-SOD), aspartate transaminase (AST), and alanine transaminase (ALT).

### Blood and liver TBA level and hepatic *fxr* and *cyp7a1* mRNA expressions

**FIGURE 4:**
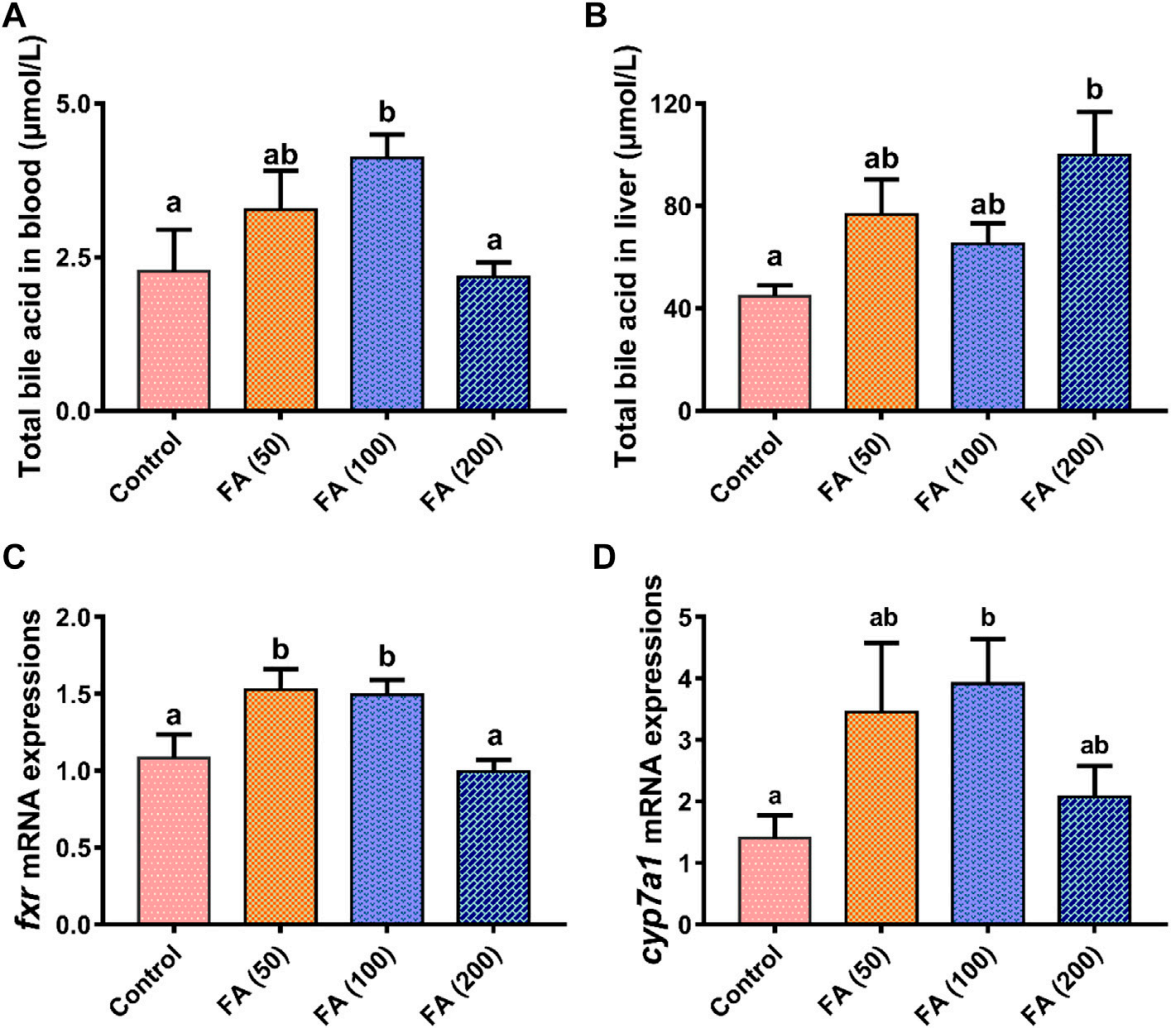
Total bile acid levels in blood **(A)** and liver **(B)** and relative mRNA expression of farnesoid X receptor (*fxr*; **(C)** and cholesterol 7α-hydroxylase (*cyp7a1*; **(D)** in the liver of grass carp fed CSM-based diets containing graded levels of ferulic acid. Control: high CSM diet; FA (50): high CSM diet +50 mg/kg ferulic acid; FA (100): high CSM diet +100 mg/kg ferulic acid; FA (200): high CSM diet +200 mg/kg ferulic acid. The results are expressed as means ± standard error of the means (*n* = 9). Means of the same parameter with different superscripts are significantly different (*p* < 0.05).

Blood (Figure 4A) and liver (Figure 4B) TBA levels in the grass carp fed CSM-based diets with graded levels of FA are illustrated in Figure 4. Compared with the control group, the dietary inclusion of 50 and 100 mg/kg FA elevated plasma TBA levels and was significantly higher in the 100 mg/kg FA group (*p* < 0.05). As dietary FA supplementation levels increased to 200 mg/kg, plasma TBA decreased, demonstrating no significant differences from the control group. Dietary FA supplementation increased liver TBA levels. The TBA levels were the highest in the fish fed with 200 mg/kg FA, significantly differing from those in the control group (*p* < 0.05).

As shown in Figure 4, the gene mRNA expression levels of farnesoid X receptor (*fxr*; Figure 4C) in the fish fed with 50 and 100 mg/kg FA were both upregulated significantly compared with the control group and the fish fed with 200 mg/kg FA (*p* < 0.05). Similarly, the liver mRNA expression of cholesterol 7α-hydroxylase (*cyp7a1*; Figure 4D) was elevated in the fish fed with 50 and 100 mg/kg FA—with this expression in the fish fed with 100 mg/kg FA being significantly higher than that in the control group (*p* < 0.05). The liver mRNA expression of *fxr* and *cyp7a1* both trended to be suppressed in the group fed with 200 mg/kg FA compared with the fish fed with lower levels of FA (50 and 100 mg/kg).

### Genes expression of intestinal tight junction proteins

**FIGURE 5:**
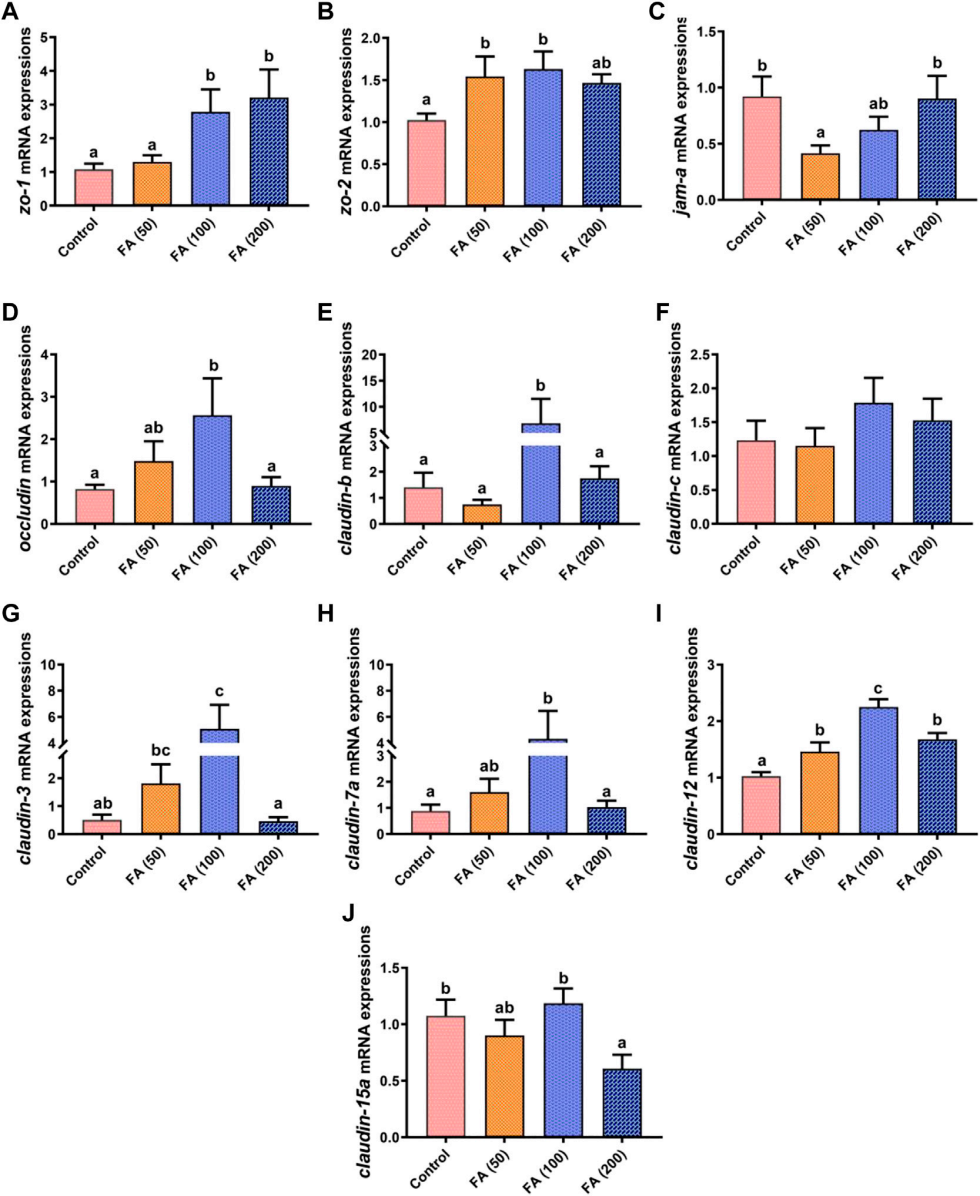
Relative mRNA expression of tight junction protein genes the zonula occludens-1 gene (*zo-1*; **(A)**, *zo-2* **(B)**, junctional adhesion molecule-A (*jam-a*; **(C)**, *occludin* **(D)**, *claudin-b* **(E)**, *claudin-c* **(F)**, *claudin-3* **(G)**, *claudin-7a* **(H)**, *claudin-12* **(I)**, and *claudin-15a* **(J)** in the intestine of juvenile grass carp fed CSM-based diets containing graded levels of ferulic acid. Control: high CSM diet; FA (50): high CSM diet +50 mg/kg ferulic acid; FA (100): high CSM diet +100 mg/kg ferulic acid; FA (200): high CSM diet +200 mg/kg ferulic acid. The results are expressed as means ± standard error of the means (*n* = 9). Means of the same parameter with different superscripts are significantly different (*p* < 0.05).

The mRNA expression of genes related to the intestinal tight junction in our grass carp is displayed in Figure 5. In general, the mRNA expression levels of zonula occludens-2 (*zo-2*; Figure 5B), *occludin* (Figure 5D), *claudin-b* (Figure 5E), *claudin-c* (Figure 5F), *claudin-3* (Figure 5G), *claudin-7a* (Figure 5H), and *claudin-12* (Figure 5I) increased gradually with increasing FA supplementation, but they (except for *claudin-12* expression) were downregulated when 200 mg/kg FA supplementation without significant difference compared with that in the control group. In detail, compared with the control group, the mRNA expression of *zo-2*, *occludin*, *claudin-b*, *claudin-3*, *claudin-7a*, and *claudin-12* in the fish fed with 100 mg/kg FA significantly increased (*p* < 0.05). Moreover, 50 mg/kg FA supplementation remarkably upregulated *zo-2* and *claudin-12* mRNA expression (*p* < 0.05). The zonula occludens-1 (*zo-1*; Figure 5A) mRNA expression increased as FA supplementation levels increased, with significant upregulation in the fish fed with 100 and 200 mg/kg FA (*p* < 0.05). However, compared with the control group, 100 and 200 mg/kg FA supplementation had no significant effect on junctional adhesion molecule-A (*jam-a*) expression (Figure 5C), but 50 mg/kg FA supplementation demonstrated remarkably downregulated *jam-a* mRNA expression (*p* < 0.05). Furthermore, the 200 mg/kg FA-supplemented diet significantly downregulated the mRNA expression of *claudin-15a* (Figure 5J) compared with the control group (*p* < 0.05).

## Discussion

In the present study, our results showed that FA supplementation (200 mg/kg) in CSM-based diets demonstrated the beneficial effects on growth performance (FBW, WGR, and SGR) and feed utilization (FCR and PER) of juvenile grass carp. As a growth stimulator, FA has been widely studied (Herrera Herrera et al., 2009; Zhu and Wakisaka, 2018; Pena-Torres et al., 2021). Yu et al. (2018) reported that dietary FA addition (100 mg/kg) significantly decreases FCR and increases WGR by 14.63% in tilapia. Moreover, according to the index of FBW, 96.33 mg/kg has been suggested as the optimal FA supplementation dose in the tilapia diet (Dawood et al., 2020), which is lower than our recommended FA dose (204 mg/kg). This difference might be attributable to differences in the species (grass carp and tilapia) and feed formula (CSM as the main protein resource in our diets) used. The mechanism of dietary FA in promoting growth performance has been elaborated by previous studies (Gorewit, 1983; Wen and Ushio, 2017; Westfall et al., 2019). The effects of FA are attributed to its structure being similar to normetanephrine, which stimulates somatotropin in the pituitary gland (Gorewit, 1983). Wen and Ushio (2017) reported that FA promotes muscle growth by activating the TOR signaling pathway. In the present study, CSM was chosen as the main protein source (384.6 g/kg feed) in the diet (containing around 400 mg/kg FG). A previous study (Wang et al., 2018) on grass carp demonstrated that a high level of FG in diets impaired growth by inhibiting *tor* mRNA expression, which partly explained the reason that our optimal FA supplementation dose was higher than that in the previous study on tilapia (Dawood et al., 2020).

When used as the main protein source in diets, CSM triggers oxidative stress and inflammation in fish because it contains high FG levels (El-Sharaky et al., 2010; Zheng et al., 2012; Bian et al., 2017). MDA, the lipid peroxidation product, is a biomarker of oxidative damage in fish (Lin et al., 2018). In studies on grass carp (Zheng et al., 2012), Ussuri catfish (*Pseudobagrus ussuriensis*) (Bu et al., 2017), and white shrimp (*Litopenaeus vannamei*) (Wang J. et al., 2020), liver MDA accumulation was found to aggravate as dietary CSM amounts increased. Yu et al. (2020) confirmed that in tilapia, FA alleviated MDA accumulation induced by oxidized fish oil in diets. In the present study, our results showed that CSM-based diets with 50 and 200 mg/kg FA supplementation significantly decreased liver MDA levels in grass carp—consistent with the previous study on tilapia (Yu et al., 2018). Our findings also showed that FA supplementation reduced blood ALT levels, reflecting improved liver health status (Banaee et al., 2007; Hoseini et al., 2016; Ghelichpour et al., 2020). Previous studies (Maurya and Devasagayam, 2010; Kim et al., 2011; Mir et al., 2018) attributed the protective effects of FA to its capacity to enhance antioxidant enzyme activities. Our previous study on blunt snout bream demonstrated that FA enhanced resistance to LPS injection-induced oxidative damage by increasing SOD, GST, GSH, and GR activities (Chen et al., 2021). Kassab et al. (2020) demonstrated that FA treatment increased GSH, SOD, CAT, GR, and glutathione peroxidase (GPX) of Wistar albino rats under CdCl_2_ stress. Similarly, in the present study, FA supplementation in a CSM-based diet increased GR and T-SOD activities and improved liver antioxidant capacity.

Studies have concluded that the composition of the diet formula considerably affects the growth performance and health status of aquatic animals by altering nutrients absorption and metabolism in the gastrointestinal tract (Trichet, 2010; Chen et al., 2020; Dawood, 2020). Therefore, intestinal health is crucial to the growth of stomachless (agastric) fish (Le et al., 2019). Studies have evaluated the effects of gossypol, the major ANFs in CSM, on the intestine health of grass carp (Wang et al., 2018; Wang K. Z. et al., 2019). They have reported that high gossypol levels in diets caused damage to the intestinal structure, aggravated intestinal inflammation, and reduced intestinal amino acid absorption capacity, all of which negatively affected growth. Tight junction proteins, such as ZOs, Occludin, JAM-A, and Claudins, are important for nutrient absorption and metabolism, which are vital for enhancing the intestinal physical barrier and inhibiting the entry of harmful molecules and microbes (Pearce et al., 2018). Studies on fish have suggested that the upregulations of *zo*, *occludin*, and *claudin* and the downregulation of *claudin-15* indicate the improvement of intestine physical barrier function (Xu et al., 2016; Wang Y. L. et al., 2019). In our study, 100 mg/kg FA supplementation in CSM-based diet significantly increased *zo-1*, *zo-2, occludin*, *claudin-b*, *claudin-3*, *claudin-7a*, and *claudin-12* mRNA expression levels and 200 mg/kg FA supplementation reduced *claudin-15a* mRNA expression. The protective effects of FA on intestinal tight junctions have been confirmed previously (Bergmann et al., 2009; Kim et al., 2013; He et al., 2016). Kim et al. (2013) confirmed that FA increased ZO-1 and Occludin protein expression levels in Caco-2 cells under tert-butyl hydroperoxide stimulation. Bergmann et al. (2009) found that FA increased *zo-1* and *claudin-4* mRNA expression but reduced that of *occludin* in a colon cell line. *In vivo*, FA was found to attenuate the effects of heat stress on the intestines by increasing *occludin* and *zo-1* mRNA expression (He et al., 2016). Taken together, these results indicate that 100 mg/kg FA supplementation in a CSM-based diet can upregulate tight junction genes and maintain the intestinal physical barrier function of grass carp. Although, no remarkable improvement in growth performance compared with the other treatment groups was found. We attributed it partly to the duration of the feeding trial (8 weeks). Therefore, potentially significant responses (such as growth performance) to FA’s graded levels were not observed probably because the experiment was terminated prematurely.

FA is mainly absorbed along the intestinal tract and then metabolized in the liver (Zhao and Moghadasian, 2008). The simultaneous enhancement of liver antioxidant capacity and intestinal barrier function, affected by FA, was observed in the present study—which consequently contributed to improving the growth of grass carp. Notably, our findings showed that FA supplementation relieved lipid deposition in fish fed CSM-based diets, possibly because of the positive effects of FA supplementation on lipid metabolism via the gut–liver axis. FA enhanced the intestinal barrier function by activating genes related to the tight junction (as we illustrated previously); this prevented the entry of harmful molecules (such as FG and LPS) into the liver through peripheral metabolism (Li et al., 2018; Lu et al., 2018; Fukawa et al., 2020). We also noted that FA supplementation increased TBA levels both in the liver and blood. Bile acid (BA), the pleiotropic signaling molecule controlling the gut–liver crosstalk (Wahlström et al., 2016; Schneider et al., 2018), plays an important role in regulating lipid metabolism (Zhou et al., 2018). Moreover, BA can activate FXR—which is crucial in BA regulation, lipid homeostasis, and barrier function of the intestine (Ding et al., 2015). Grass carp fed the FXR agonist GW4064 was reported to demonstrate low hepatopancreatic TG levels, but the FXR inhibitor glycine-β-muricholic acid was reported to reverse these effects (Tian et al., 2021). Moreover, high TG, blood V-LDL, and liver TC levels as well as grave liver steatosis can be noted in FXR-knockout mice (Sinal et al., 2000). CYP7A1, as the first rate-limiting enzyme in BA synthesis, is an important way of BA feedback regulation of BA homeostasis (Chiang and Ferrell, 2020). Consistent with the aforementioned studies, the current study demonstrated that *fxr* and *cyp7a1* mRNA expression levels were high in the fish fed with 50 and 100 mg/kg FA, indicating high blood and liver TBA levels. In general, we found that FA could regulate lipid metabolism via regulating BA secretion.

## Conclusion

**FIGURE 6:**
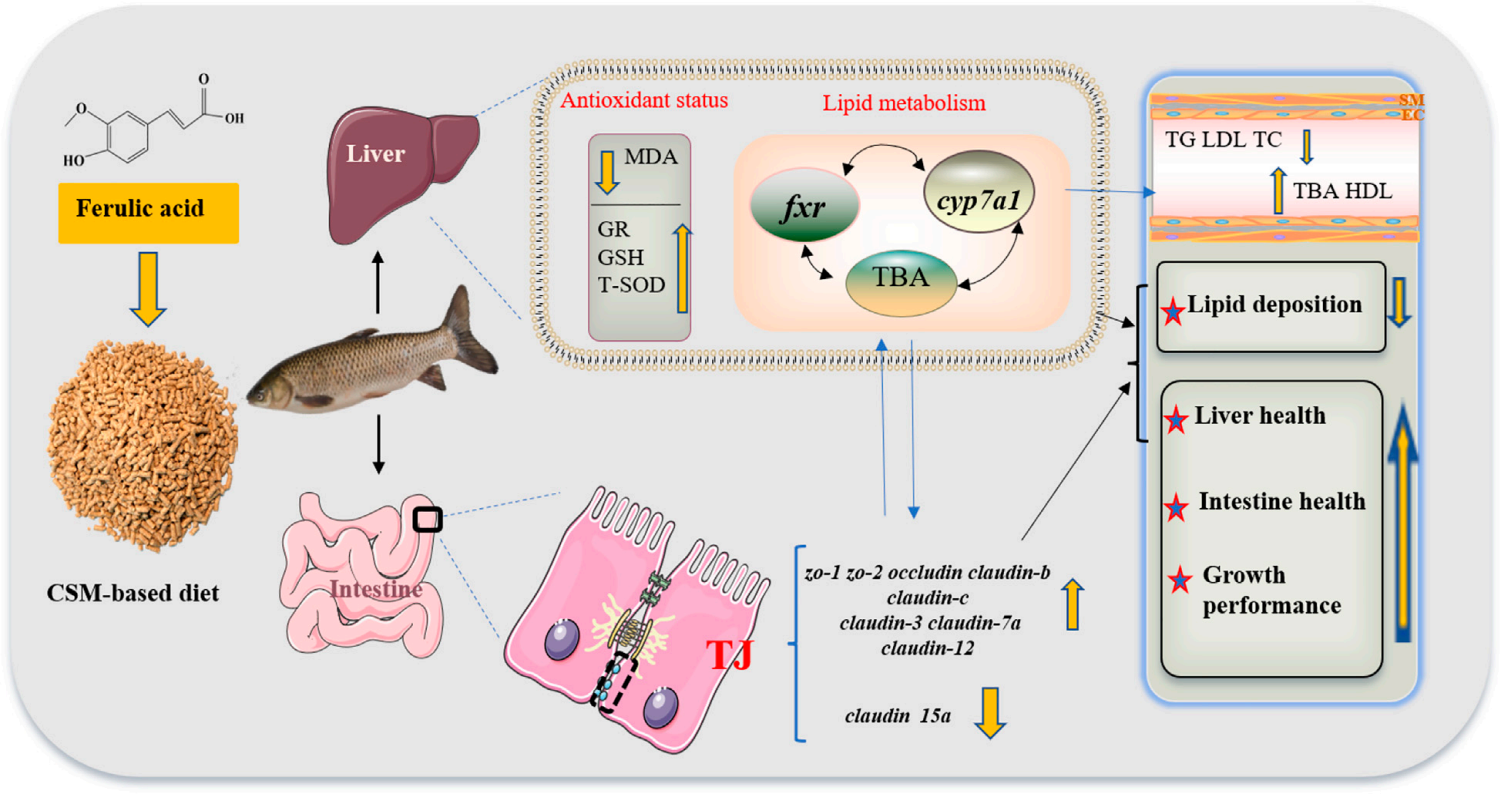
Schematic of the beneficial effects of optimal FA supplementation in CSM-based diets on lipid metabolism, antioxidant status, and growth performance of juvenile grass carp. Abbreviations: cottonseed meal (CSM), malondialdehyde (MDA), glutathione reductase (GR), glutathione (GSH), total superoxide dismutase (T-SOD), farnesoid X receptor (*fxr*); cholesterol 7α-hydroxylase (*cyp7a1*), total bile acid (TBA), zonula occludens-1/2 (*zo-1/2*), triglyceride (TG), low-density lipoprotein (LDL), total cholesterol (TC), tight junction (TJ), and high-density lipoprotein (HDL).

As shown in Figure 6, FA supplementation in CSM-based diet could activate genes related to the tight junction to maintain intestinal physical barrier function and improve liver antioxidant capacity. It could also reduce whole-body lipid deposition, and alter plasma lipid parameters by regulating BA secretion, while the regulation mechanism needs further study. Therefore, the use of FA as a feed additive may boost fish growth performance and CSM utilization.

## Data Availability

The original contributions presented in the study are included in the article/Supplementary Material, further inquiries can be directed to the corresponding authors.
